
JOURNAL INFORMATION
==============================
NLM Title Abbreviation: Orthop Surg
Journal ID: Orthop Surg
NLM Journal ID: 101501666
Journal ID: OS

Orthopaedic Surgery

ISSN: 1757-7853
EISSN: 1757-7861
Publisher: Wiley

ARTICLE INFORMATION
==============================
PMCID: PMC11474638
DOI: 10.1111/os.13785
Article ID: OS13785
Article version: 1
Subjects: Issue Information, Issue Information

Issue Information

Electronic publication date: 2024 May 1
Publication date: 2024 May
Volume: 16
Issue: 5
First page: 1017
Last page: 1018
Copyright: © 2024 The Authors. Orthopaedic Surgery published by Tianjin Hospital and John Wiley & Sons Australia, Ltd.
License: This is an open access article under the terms of the http://creativecommons.org/licenses/by/4.0/ License, which permits use, distribution and reproduction in any medium, provided the original work is properly cited.
License URL: https://creativecommons.org/licenses/by/4.0/

Figure count: 0
Table count: 0
Page count: 1

source-schema-version-number: 2.0
cover-date: May 2024
details-of-publishers-convertor: Converter:WILEY_ML3GV2_TO_JATSPMC version:6.4.9 mode:remove_FC converted:15.10.2024

==============================

Volume 16 • Issue 5 • May 2024 • ISSN 1757-7861

### Orthopaedic Surgery

Open Access

REVIEW ARTICLE

1019
DOES PROXIMAL ADDUCTOR CANAL BLOCK PROVIDE BETTER ANALGESIC EFFICACY THAN DISTAL ADDUCTOR CANAL BLOCK IN PATIENTS UNDERGOING KNEE ARTHROPLASTY: A SYSTEMATIC REVIEW AND META-ANALYSIS OF RANDOMIZED CONTROLLED TRIALS

CLINICAL ARTICLES

1034
COMPARISON OF THE CLINICAL OUTCOMES BETWEEN ALL-INSIDE AND STANDARD TECHNIQUE IN ANTERIOR CRUCIATE LIGAMENT RECONSTRUCTION WITH 6-STRAND HAMSTRING TENDON AUTOGRAFT

1042
COMPARISON OF THE MEDIUM-TERM OUTCOMES OF ANTERIOR LUMBAR DISCECTOMY AND FUSION WITH MINIMALLY INVASIVE TRANSFORAMINAL LUMBAR INTERBODY FUSION: A RETROSPECTIVE COHORT STUDY

1051
ASSOCIATION OF HANDGRIP STRENGTH WITH HIP FRACTURE AND FALLS IN COMMUNITY-DWELLING MIDDLE-AGED AND OLDER ADULTS: A 4-YEAR LONGITUDINAL STUDY

1064
MODIFIED PERCUTANEOUS ENDOSCOPIC INTERLAMINAR DISCECTOMY THROUGH THE NEAR-SPINOUS PROCESS APPROACH FOR L4/5 DISC HERNIATION: A RETROSPECTIVE CLINICAL STUDY

1073
THE NEW DOUBLE-ROW BANKART REPAIR RECOVERED SHOULDER STABILITY WITHOUT EXCESSIVE MOTION LIMITATION: A CASE–CONTROL STUDY WITH SINGLE-ROW BANKART REPAIR

1079
COMPARISON OF CLINICAL EFFICACY BETWEEN ARTHROSCOPIC AND OPEN SURGERY FOR OGDEN TYPE 1–2 PERONEAL TENDON DISLOCATION

1089
EFFECT OF PATELLAR MORPHOLOGY ON THE RISK OF OSTEOCHONDRAL FRACTURE AFTER PATELLAR DISLOCATION: A CROSS-SECTIONAL STUDY

1101
A NEW METHOD TO PREDICT POSTOPERATIVE STEM ANTEVERSION IN TOTAL HIP ARTHROPLASTY FOR DEVELOPMENTAL DYSPLASIA OF THE HIP

1109
CLINICAL APPLICATION AND CURATIVE EFFECT ANALYSIS OF POSTURAL AWARENESS SURGICAL TOOL ASSISTED NAIL PLACEMENT IN ADOLESCENT IDIOPATHIC SCOLIOSIS

1117
A MODIFIED ARTHROSCOPIC TRIPLE-ROW REPAIR TECHNIQUE FOR L-SHAPED DELAMINATED ROTATOR CUFF TEARS

1127
COMPARISON OF OUTCOMES IN OBESE PATIENTS AFTER TOTAL KNEE ARTHROPLASTY WITH NEUTRAL OR MILD VARUS: A RETROSPECTIVE STUDY WITH 8-YEAR FOLLOW-UP

1134
CLINICAL EFFICACY OF UNILATERAL DUAL-CHANNEL ENDOSCOPIC LUMBAR INTERBODY FUSION FOR LUMBAR SPONDYLOLISTHESIS WITH SPINAL SCOLIOSIS

1143
CHANGES IN PATELLAR HEIGHT AND TIBIAL POSTERIOR SLOPE AFTER BIPLANAR HIGH TIBIAL OSTEOTOMY WITH COMPUTER-DESIGNED PERSONALIZED SURGICAL GUIDES: A RETROSPECTIVE STUDY

1153
CLINICAL OUTCOMES FOLLOWING INCOMPLETE ARTHROSCOPIC ILIOTIBIAL BAND RELEASE FOR EXTERNAL SNAPPING HIP SYNDROME: AN OBSERVATIONAL STUDY

1160
THE NEGATIVE IMPACTS OF SARCOPENIA ON PRIMARY TOTAL KNEE ARTHROPLASTY UNDER THE ENHANCED RECOVERY AFTER SURGERY PROTOCOL

1168
COMPARISON OF ALIGNMENT ACCURACY AND CLINICAL OUTCOMES BETWEEN A CT-BASED, SAW CUTTING ROBOTIC SYSTEM AND A CT-FREE, JIG-GUIDED ROBOTIC SYSTEM FOR TOTAL KNEE ARTHROPLASTY

RESEARCH ARTICLES

1175
MECHANISM OF FAT MASS AND OBESITY-RELATED GENE-MEDIATED HEME OXYGENASE-1 M6A MODIFICATION IN THE RECOVERY OF NEUROLOGICAL FUNCTION IN MICE WITH SPINAL CORD INJURY

1187
RELIABILITY AND ACCURACY OF THE OUTERBRIDGE CLASSIFICATION IN STAGING OF CARTILAGE DEFECTS

1196
THREE-DIMENSIONAL MAPPING ANALYSIS OF TALUS FRACTURES AND DEMONSTRATION OF DIFFERENT SURGICAL APPROACHES FOR TALUS FRACTURES

1207
COMPARISON OF TWO SURGICAL APPROACHES FOR PERIACETABULAR OSTEOTOMY: A RETROSPECTIVE STUDY OF PATIENTS WITH DEVELOPMENTAL DYSPLASIA OF THE HIP

1215
A CONTROLLED VARIABLE STUDY OF THE BIOMECHANICAL PROPERTIES OF THE PROXIMAL FEMUR BEFORE AND AFTER CANCELLOUS BONE REMOVAL

1230
HOMEOPATHIC ANKLE DISLOCATION FOR TREATMENT OF UNSTABLE TRIMALLEOLAR FRACTURES INVOLVING POSTERIOR DIE-PUNCH FRAGMENT: A RETROSPECTIVE COHORT STUDY

OPERATIVE TECHNIQUES

1239
APPLICATION OF PIEZOSURGERY IN REVISION SURGERY THROUGH POSTERIOR APPROACH FOR INFECTION AFTER PERCUTANEOUS VERTEBRAL AUGMENTATION: TECHNIQUE NOTE WITH CASE SERIES

1246
ROBOT-ASSISTED PERCUTANEOUS RADIOFREQUENCY ABLATION FOR THE TREATMENT OF OSTEOID OSTEOMAS

CORRECTIONS

1252
CORRECTION TO “CORRELATION BETWEEN CONDITIONAL APPROVAL AND CUSTOMIZED BONE IMPLANT DEVICES”

1253
CORRECTION TO “EPIDEMIOLOGICAL STUDY OF TIBIAL PLATEAU FRACTURES COMBINED WITH INTERCONDYLAR EMINENCE FRACTURES”

WILEY

CHINESE ORTHOPAEDIC ASSOCIATION
中华医学会骨科学分会

View this journal online at wileyonlinelibrary.com/journal/os

### Orthopaedic Surgery

**Honorary Editor-in-Chief**
Guixing Qiu, *Beijing, China*

**Editor-in-Chief**
Xinlong Ma, *Tianjin, China*

**Associate Editors**
Cyrus Cooper, *Southampton, UK*
Benjamin G. Domb, *Des Plaines, USA*
Qing Jiang, *Nanjing, China*
Baoge Liu, *Beijing, China*
Zhuojing Luo, *Xi'an, China*
Luigi Meccariello, *Moiano, Italy*
Ling Qin, *Hong Kong, China*
Qi Yin, *Liverpool, UK*

**Editorial Board**
Elisa Belluzzi, *Padua, Italy*
Carlo Biz, *Padua, Italy*
Mary L Bouxsein, *Boston, USA*
Richard E. Buckley, *Calgary, Canada*
Zhengdong Cai, *Shanghai, China*
Wei Chai, *Beijing, China*
Bohua Chen, *Qingdao, China*
Liang Chen, *Soochow, China*
Yanxi Chen, *Shanghai, China*
Pramod Devkota, *Patan, Nepal*
Meng Fan, *Tianjin, China*
Haoyu Feng, *Taiyuan, China*
Maoqi Gong, *Beijing, China*
Yong Hai, *Beijing, China*
Wei Huang, *Chongqing, China*
Baochao Ji, *Urumqi, China*
Ayhan Kanat, *Rize, Turkey*
Kenny Kwan, *Hong Kong, China*
Sallie Lamb, *Exeter, UK*
Lifeng Lao, *Shanghai, China*
Jung-Hee Lee, *Seoul, Korea*
Guanghua Lei, *Changsha, China*
Guoan Li, *Newton, USA*
Hui Li, *Tianjin, China*
Jianmin Li, *Jinan, China*
Weishi Li, *Beijing, China*
Jianhao Lin, *Beijing, China*
Fanxiao Liu, *Munich, Germany*
Ruben Lostado, *La Rioja, Spain*
Songchen Lyu, *Harbin, China*
Weihu Ma, *Ningbo, China*
Xin Ma, *Shanghai, China*
Masaya Nakamura, *Tokyo, Japan*
Muthu Sathish, *Velayuthampalayam, India*
Wolfgang Senker, *Linz, Austria*
Sancar Serbest, *Boston, USA*
Cailiang Shen, *Hefei, China*
Bin Shen, *Chengdu, China*
Huiyong Shen, *Shenzhen, China*
Jianxiong Shen, *Beijing, China*
Puyi Sheng, *Guangzhou, China*
Junying Sun, *Soochow, China*
Shui Sun, *Jinan, China*
YasuhikoTakegami, *Nagoya, Japan*
Sakae Tanaka, *Tokyo, Japan*
Xiaodong Tang, *Beijing, China*
Fei Wang, *Shijiazhuang, China*
Weijun Wang, *Nanjing, China*
Xu Wang, *Shanghai, China*
Zheng Wang, *Beijing, China*
Jidapa Wongcharoenwatana, *Bangkok, Thailand*
Chi-Chuan Wu, *Taipei, China*
Nan Wu, *Beijing, China*
Zhihong Wu, *Beijing, China*
Yayi Xia, *Lanzhou, China*
Jianru Xiao, *Shanghai, China*
Zhongyu Xie, *Shenzhen, China*
Dan Xing, *Beijing, China*
Weiguo Xu, *Tianjin, China*
Peng Xu, *Xi 'an, China*
CaoYang, *Wuhan, China*
Pei Yang, *Xi 'an, China*
Zhaoming Ye, *Hangzhou, China*
Xiuchun Yu, *Jinan, China*
Bin Yu, *Guangzhou, China*
Yi Zeng, *Chengdu, China*
Dianying Zhang, *Beijing, China*
Hong Zhang, *Texas, USA*
Weibin Zhang, *Shanghai, China*
Xianlong Zhang, *Shanghai, China*
Jianguo Zhang, *Beijing, China*
Bin Zhao, *Taiyuan, China*
Jie Zhao, *Shanghai, China*
Yu Zhao, *Beijing, China*
Zongke Zhou, *Chengdu, China*
Yue Zhu, *Shenyang, China*
Zezhang Zhu, *Nanjing, China*

**Address for Editorial Correspondence:** Editorial Board of *Orthopaedic Surgery*, Tianjin Hospital, 122 Munan Road, Tianjin 300050, China (email: orthopaedicsurgery@126.com; telephone: +86 22 2833 4734; fax: +86 22 2824 1184).

**Disclaimer:** The Publisher, Tianjin Hospital and Editors cannot be held responsible for errors or any consequences arising from the use of information contained in this journal; the views and opinions expressed do not necessarily reflect those of the Publisher, Tianjin Hospital and Editors, neither does the publication of advertisements constitute any endorsement by the Publisher, Tianjin Hospital and Editors of the products advertised.

**Copyright © 2024 The Authors. Orthopaedic Surgery published by Tianjin Hospital and John Wiley & Sons Australia, Ltd**

**Copyright and Copying (in any format)**
Copyright © 2024 The Authors. Orthopaedic Surgery published by Tianjin Hospital and John Wiley & Sons Australia, Ltd. All rights reserved. No part of this publication may be reproduced, stored or transmitted in any form or by any means without the prior permission in writing from the copyright holder. Authorization to copy items for internal and personal use is granted by the copyright holder for libraries and other users registered with their local Reproduction Rights Organisation (RRO), e.g. Copyright Clearance Center (CCC), 222 Rosewood Drive, Danvers, MA 01923, USA (www.copyright.com), provided the appropriate fee is paid directly to the RRO. This consent does not extend to other kinds of copying such as copying for general distribution, for advertising or promotional purposes, for republication, for creating new collective works or for resale. Permissions for such reuse can be obtained using the RightsLink "Request Permissions" link on Wiley Online Library. Special requests should be addressed to: permissions@wiley.com

For submission instructions, subscription and all other information visit: http://wileyonlinelibrary.com/journal/os

ISSN 1757-7861 (Online)

OS.JEB.May24
